# Recognizing the value of software: a software citation guide

**DOI:** 10.12688/f1000research.26932.2

**Published:** 2021-01-12

**Authors:** Daniel S. Katz, Neil P. Chue Hong, Tim Clark, August Muench, Shelley Stall, Daina Bouquin, Matthew Cannon, Scott Edmunds, Telli Faez, Patricia Feeney, Martin Fenner, Michael Friedman, Gerry Grenier, Melissa Harrison, Joerg Heber, Adam Leary, Catriona MacCallum, Hollydawn Murray, Erika Pastrana, Katherine Perry, Douglas Schuster, Martina Stockhause, Jake Yeston

**Affiliations:** 1 University of Illinois at Urbana-Champaign, Urbana, IL, USA; 2 EPCC, University of Edinburgh, Edinburgh, UK; 3 University of Virginia, Charlottesville, VA, USA; 4 American Astronomical Society, Washington, DC, USA; 5 American Geophysical Union, Washington, DC, USA; 6 Harvard-Smithsonian Center for Astrophysics, Cambridge, MA, USA; 7 Taylor & Francis Group, Oxford, UK; 8 GigaScience Press, BGI Hong Kong, Hong Kong, Hong Kong; 9 Elsevier, Amsterdam, The Netherlands; 10 Crossref, Lynnfield, MA, USA; 11 DataCite, Hannover, Germany; 12 American Meteorological Society, Boston, MA, USA; 13 Publishing Technology, IEEE, Piscataway, NJ, USA; 14 Production, eLife, Cambridge, UK; 15 PLOS, San Francisco, CA, USA; 16 Oxford University Press, Oxford, UK; 17 Open Science, Hindawi, London, UK; 18 F1000Research, London, UK; 19 Springer Nature, New York, NY, USA; 20 Product Management, Wiley, Boston, MA, USA; 21 National Center for Atmospheric Research, Boulder, CO, USA; 22 German Climate Computing Center (DKRZ), Hamburg, Germany; 23 AAAS, Washington, DC, USA

**Keywords:** Software citation, publishing, scholarly communication, guidelines, bibliometrics

## Abstract

Software is as integral as a research paper, monograph, or dataset in terms of facilitating the full understanding and dissemination of research. This article provides broadly applicable guidance on software citation for the communities and institutions publishing academic journals and conference proceedings. We expect those communities and institutions to produce versions of this document with software examples and citation styles that are appropriate for their intended audience. This article (and those community-specific versions) are aimed at authors citing software, including software developed by the authors or by others. We also include brief instructions on how software can be made citable, directing readers to more comprehensive guidance published elsewhere. The guidance presented in this article helps to support proper attribution and credit, reproducibility, collaboration and reuse, and encourages building on the work of others to further research.

Software is as integral as a research paper, monograph, or dataset in terms of facilitating the full understanding and dissemination of research. Books and journal articles have long benefited from an infrastructure that makes them easy to cite, a key element in the process of research and academic discourse in all disciplines. We believe that software (including computational code, scripts, models, notebooks and libraries) should be cited in the same way that other sources of information, such as articles and books, are cited.

Citing software helps further research and provides the means for other researchers to access software in order to:

- support proper attribution and credit (similar to that of papers, data, etc.);
- enable peer-review, validation, and reproducibility of findings;
- support collaboration and reuse; and
- encourage building on the work of others.

Software citation elevates software to the level of a first-class object in the digital scholarly ecosystem, consistent with its immense actual present-day significance.

FORCE11 has been developing guidance for software citation. The Software Citation Principles ( Smith *et al.*, 2016) were written to encourage broad adoption of a consistent policy for software citation across disciplines and venues. The Software Citation Checklist for Authors ( Chue Hong *et al.*, 2019a) and Software Citation Checklist for Developers ( Chue Hong *et al.*, 2019b) provide more practical information for those seeking to improve their practice. This work has been influenced by prior work on Data Citation ( Data Citation Synthesis Group, 2014), while recognizing that software is not the same as data in the context of citation ( Katz *et al.*, 2016).

## Software citation essentials

This article is aimed at authors citing software. This includes software developed by others, as well as software developed by any or all of the authors. Making software citable is a critical developer-led step, which is briefly detailed in the next subsection, "Making Software Citable".

The use of persistent identifiers (PIDs) and core descriptive metadata are essential elements of software citation. This is because they are the mechanism used to index and track citations. We recognise that the challenges associated with software deposit and publication vary across disciplines, and we encourage research communities to develop citation systems that work well for them. We also recognise that the citation style formats used vary between disciplines and journals. Independent of the style of any citation, we recommend certain essential metadata elements should always be captured.

There are multiple use cases for citing software. These include referring to the software used in deriving the results of an article or discussing algorithms, general features, or concepts provided by a piece of software. If you used the software directly in the research described in your article (e.g., in the Methods section), then we recommend citing the specific version used (and the authors and publication date for that version). When discussing software more broadly, we recommend citing the software as a concept (project).

Our recommended format for software citation is to ensure the following information is provided as part of the reference:

- *Creator(s):* the authors or project that developed the software.
- *Title:* the name of the software.
- *Publication venue:* the publication venue of the software, preferentially, an archive or repository that provides persistent identifiers.
- *Date:* the date the software was published. This is the date associated with a release or version of the software, or “n.d.” if the date is unknown.
- *Identifier:* a resolvable pointer to the software, preferentially, a PID that resolves to a landing page containing descriptive metadata about the software, similar to how a Digital Object Identifier (DOI) for a paper that points to a page about the paper rather than directly to a representation of the paper, such as the PDF. DOIs are preferable, and other examples of PIDs include Handles, RRIDs, ASCL IDs, swMath IDs, Software Heritage IDs, ARKs, etc. If there is no PID for the software, a URL to where the software exists may be the best identifier available.

It may also be desirable, and depending upon the publisher, may be required, to include information about two optional properties (as appropriate):

- *Version:* the identifier for the version of the software being referenced. If the version is unidentified or unknown, the date of access should be used.
- *Type:* some citation styles (e.g., APA), require a bracketed description of the citation (e.g., Computer software) to be included.

If an article exists that describes the software, it should be cited as an additional reference, as well as citing the software itself. Do not cite the article instead of the software.

## Making software citable

Authors should consult the Software Citation Checklist for Developers ( Chue Hong *et al.,* 2019b) for information on how to obtain a PID or choose a software license for software they have developed. That document contains a set of steps that developers can take to ensure that they are following good practices. We strongly recommend that journals provide such information to their authors, either by referring to that document, or using text from it or similar text. Example guidance would include instructing authors to version their software, choose a license for their software, perhaps by linking to the information at choosealicense.org, record metadata about the software as part of the repository, deposit their software in a preservation repository that provides a PID, and advertise the recommended citation in the repository. In particular, guidance should explicitly mention that Creative Commons licenses (including CC-BY) must not be used for software, and an open source license should be used.

## Software citation examples

The following examples show how software can be cited in one common citation style, APA. The general format for downloaded software, from Section 10.10 of (2020) Publication Manual of the American Psychological Association (Seventh Edition) is:

- Developer, A. A., Developer, B. B., & Developer, C. C. (yyyy) ^1^. *Title of the software: Subtitle* (Version #.#) ^2^ [Computer software] ^3^. Publisher ^4^, https://URL ^5^

If no version number or version string exists, we (the FORCE11 Software Citation Implementation Working Group) modify this to:

- Developer, A. A., Developer, B. B., & Developer, C. C. (yyyy). *Title of the software: Subtitle* [Computer software]. Archive Name. Retrieved Month dd, yyyy, from https://URL

The following are examples of software citations.

Ideal citations to the specific version of the software, where all recommended information is present (the first demonstrates a large author list; the second demonstrates a project team as the author):

- Coon, E., Berndt, M., Jan, A., Svyatsky, D., Atchley, A., Kikinzon, E., Harp, D., Manzini, G., Shelef, E., Lipnikov, K., Garimella, R., Xu, C., Moulton, D., Karra, S., Painter, S., Jafarov, E., & Molins, S. (2020, March 25). *Advanced Terrestrial Simulator (ATS) v0.88* (Version 0.88) [Computer software]. Zenodo. https://doi.org/10.5281/zenodo.3727209
- Lab For Exosphere And Near Space Environment Studies. (2019, March 20). *lenses-lab/LYAO_RT-2018JA026426: Original Release* (Version 1.0.0) [Computer software]. Zenodo. https://doi.org/10.5281/zenodo.2598836

Citation referencing software that is preserved in a software archive (e.g. Software Heritage) ^6^:

- Delebecque, F., Gomez, C., Goursat, M., Nikoukhah, R., Steer, S., & Chancelier, J.-P. (1994). *Scilab* (Version 1.1) [Computer software]. Software Heritage, swh:1:dir:1ba0b67b5d0c8f10961d878d91ae9d6e499d746a;origin= https://hal.archives-ouvertes.fr/hal-02090402
- Di Cosmo, R. & Danelutto, M. (2020). *The Parmap library: Core mapping routine* (Version 1.1.1) [Computer software]. Software Heritage, swh:1:cnt:43a6b232768017b03da934ba22d9cc3f2726a6c5;lines=192-228;origin= https://github.com/rdicosmo/parmap

A citation for software that does not have a PID but does have a version and identifier (URL), where authorship is assigned to the project as a whole:

- Dataverse Project (2020). *Dataverse* (Version 4.20) [Computer software] https://github.com/IQSS/dataverse/releases/tag/v4.20

A citation for software where there is no version identified and where the publishing date is unknown:

- Thomas, J. & Daujotas, G. ^7^ (n.d.). *is-thirteen* [Computer software]. GitHub. Retrieved June 17, 2020 from https://github.com/jezen/is-thirteen

A citation for a software concept (all versions):

- BLAS team (n.d.), *BLAS (Basic Linear Algebra Subprograms)* [Computer software]. Netlib. http://www.netlib.org/blas/

A citation for software where little information is available, perhaps where only the executable program is available. For commercial software, a link to information about availability for purchase is helpful, as shown in the example below.

- IBM Corp. (2017). *IBM SPSS Statistics for Windows* (Version 25.0) [Computer software]. IBM Corp. https://www.ibm.com/products/spss-statistics

### In-text referencing

Two examples of how the citations above would be referenced in the text of a paper according to APA style ^8^, the first in the methodology section and the second in a related work section:

- We used version 0.88 of Advanced Terrestrial Simulator (Coon *et al.*, 2019) and version 25.0 of IBM SPSS Statistics for Windows (IBM Corp., 2017) to carry out the analysis of the data in this paper.
- In the field of bibliometrics, a different approach is taken by BLAS (BLAS team, n.d).

## Usage note

This document provides generic guidance about software citation for the communities and institutions publishing academic journals and conference proceedings. We expect those communities and institutions to produce different versions of this document with software examples and citation styles that are appropriate for their intended audience. We request that those documents refer back to (or cite) this one. This document can be cited (in APA 7th Ed. style) as:

Katz, D. S., Chue Hong, N. P., Clark T., Muench, A., Stall, S., Bouquin, D., Cannon, M., Edmunds, S., Faez, T., Farmer, R., Feeney, P., Fenner, M., Friedman, M., Grenier, G., Harrison, M., Heber, J., Leary, A., MacCallum, C., Murray, H., … Yeston, J. (2020) *Recognizing the value of software: a software citation guide.* F1000 Research. https://doi.org/10.12688/f1000research.26932.2

## Data availability

No data is associated with the article.

## Footnotes

^1^The year is required, or “n.d.” if not identifiable.

^2^The version is optional but preferred. Note that the version may be a token/string that is not a semantic version ( https://semver.org/) and that must be exactly preserved, such as a commit hash (e.g., a149dbc00fe8b0e8260f7c2d39c77692683e7fa4), a semi-numeric tagged release (e.g., v0.4-alpha01), or date string (e.g., 2020-02-20).

^3^APA style includes additional information that is helpful for software citation (e.g. it requires the [Computer software] bracketed description). Although this is not part of our guidance above, we recommend following APA style and including these elements. Other styles may not use this extra information.

^4^If the software is downloaded or if the developer is the same as the publisher, the publisher name is omitted.

^5^In APA style, the URL is used for both URLs and DOIs or other PIDs, e.g., a DOI is expressed as https://doi.org/DOI.

^6^This example is analogous to citing the preserved version of a webpage on archive.org, rather than the webpage directly.

^7^The README for the *is-thirteen* software says “A helpful tool by Jezen Thomas with helpful help from Gytis Daujotas and many fine folk.”; therefore our citation tries to take the developers intentions around authorship into account.

^8^American Psychological Association. (2020). *Publication manual of the American Psychological Association* (7th ed.). American Psychological Association. https://doi.org/10.1037/0000165-000
