# Supplementary material for: Recognizing the value of software: a software citation guide
Source: F1000Res. 2021 Jan 12;9:1257. Originally published 2020 Oct 19. [Version 2] doi: 10.12688/f1000research.26932.2 (PMC7805487; doi:10.12688/f1000research.26932.2)
Supplement: Peer review history

# Peer review: Reviewer response for version 2

**DOI:** 10.5256/f1000research.47168.r77167

**Published:** 2021-01-13

**Authors:** Ludo Waltman

**Affiliations:** 1 Centre for Science and Technology Studies (CWTS), Leiden University, Leiden, The Netherlands

I am happy with the revised version of this article. I don't have any requests for further changes.

Is the rationale for developing the new method (or application) clearly explained?

Yes

Is the description of the method technically sound?

Yes

Are the conclusions about the method and its performance adequately supported by the findings presented in the article?

Yes

If any results are presented, are all the source data underlying the results available to ensure full reproducibility?

Yes

Are sufficient details provided to allow replication of the method development and its use by others?

Yes

Reviewer Expertise:

Scientometrics, quantitative science studies, open science

I confirm that I have read this submission and believe that I have an appropriate level of expertise to confirm that it is of an acceptable scientific standard.

# Peer review: Reviewer response for version 1

**DOI:** 10.5256/f1000research.29749.r75320

**Published:** 2020-12-07

**Authors:** Gianmaria Silvello

**Affiliations:** 1 Department of Information Engineering, University of Padua, Padua, Italy

This paper presents an overview of software citation detailing state of the art and providing some indications about how software should be cited in different contexts. 

 There is no innovative method presented, but rather this is a set of community-driven guidelines that can be really useful as a starting point to provide adequate software citations.

 I am not sure this paper is a good fit as a method article for this journal, but it is a good fit for the journal. To me, it can be something in-between a method article and an opinion paper.

 What can be considered missing from this paper are considerations or references to transitive citations, which are central for software citation.  Nevertheless, this topic may be out of scope for a contribution like this one. 

 Anyway, the paper is well-written, and to me, it can be published as-is provided that it is clear that no major innovative contribution is described or new insight is presented.

Is the rationale for developing the new method (or application) clearly explained?

Yes

Is the description of the method technically sound?

Yes

Are the conclusions about the method and its performance adequately supported by the findings presented in the article?

Yes

If any results are presented, are all the source data underlying the results available to ensure full reproducibility?

No source data required

Are sufficient details provided to allow replication of the method development and its use by others?

Yes

Reviewer Expertise:

Databases, Data citation, Information retrieval and Digital Libraries,

I confirm that I have read this submission and believe that I have an appropriate level of expertise to confirm that it is of an acceptable scientific standard.

# Response

**Published:** 2021-01-04

**Authors:** Daniel S. Katz

**Affiliations:** University of Illinois at Urbana-Champaign, USA

Thank you for your review and suggestions. In our newly submitted revision, we considered your point

- What can be considered missing from this paper are considerations or references to transitive citations, which are central for software citation.  Nevertheless, this topic may be out of scope for a contribution like this one. 

but we feel this is indeed out of scope for this paper.

# Peer review: Reviewer response for version 1

**DOI:** 10.5256/f1000research.29749.r73368

**Published:** 2020-11-17

**Authors:** Ludo Waltman

**Affiliations:** 1 Centre for Science and Technology Studies (CWTS), Leiden University, Leiden, The Netherlands

This is a very useful contribution. I have some minor comments.

 Discussions about software citation and data citation are closely related. I would therefore find it helpful to read something about the way in which the guidance on software citation provided in this document relates to standards for data citation. It seems important that standards for software citation and data citation are consistent as much as possible.

 The title of the contribution (“The importance of software citation”) suggests that the contribution focuses on arguing for the importance of software citation. However, as explained in the abstract, the focus in fact is on providing “broadly applicable guidance on software citation”. My suggestion therefore is to revise the title. An alternative title for instance could be “How to cite software?”.

 “We recommend citing the specific version used (and the authors and publication date for that version) if you used it directly in the research described in your publication (e.g., the Methods section). We recommend citing the software concept (project) if you are referencing the software elsewhere in your paper.”: I don’t fully understand the distinction that is made in these two sentences. The authors seem to have in mind a distinction between citing software because it is used directly in a research project and citing software for other reasons. I would like to know more about what other reasons for citing software the authors have in mind and why they believe citations should be made in different ways in the two situations they distinguish.

 “If a published article exists that describes the software, it should be cited as an additional reference.”: The motivation for this recommendation is not clear to me. The authors seem to give special treatment to published articles, by which I assume they have in mind articles published in scholarly journals. I find this questionable. Suppose we have two pieces of software. Software A is documented in a two-page article published in a scholarly journal. Software B is documented in a comprehensive report made available in GitHub. Why should the article documenting software A be cited, while the report documenting software B does not need to be cited? Note that the article documenting software A probably cannot be updated, and the article is therefore likely to provide an outdated description of the software. The report documenting software B can be updated and therefore is likely to offer an up-to-date description of the software.

 “Hardware is important, but we have initially chosen not to overload software citations with hardware requirements directly. This might be better done through linkage between DOIs.”: I don’t understand these two sentences. Some additional explanation would be helpful.

Is the rationale for developing the new method (or application) clearly explained?

Yes

Is the description of the method technically sound?

Yes

Are the conclusions about the method and its performance adequately supported by the findings presented in the article?

Yes

If any results are presented, are all the source data underlying the results available to ensure full reproducibility?

Yes

Are sufficient details provided to allow replication of the method development and its use by others?

Yes

Reviewer Expertise:

Scientometrics, quantitative science studies, open science

I confirm that I have read this submission and believe that I have an appropriate level of expertise to confirm that it is of an acceptable scientific standard, however I have significant reservations, as outlined above.

# Response

**Published:** 2021-01-04

**Authors:** Daniel S. Katz

**Affiliations:** University of Illinois at Urbana-Champaign, USA

Thank you for your comments.

 We have just submitted a revised version that adds some additional description to explain the item about the software's publication date, as you requested.

 Regarding your second point, suggesting that discuss the recent CDUR work, we believe this paper has a fairly narrow focus, and that a future expanded or follow-on paper would be a better place to compare with that work, as well as much other related work.

# Response

**Published:** 2021-01-04

**Authors:** Daniel S. Katz

**Affiliations:** University of Illinois at Urbana-Champaign, USA

Thank you very much for your careful reading and useful comments and suggestions.  We have just submitted a revised version of the paper, which has the following changes made in response:

- Discussions about software citation and data citation are closely related. I would therefore find it helpful to read something about the way in which the guidance on software citation provided in this document relates to standards for data citation. It seems important that standards for software citation and data citation are consistent as much as possible.

We've added a sentence at the end of this paragraph to recognize the connection to work on data citation, and to point readers to references for more information.

- The title of the contribution (“The importance of software citation”) suggests that the contribution focuses on arguing for the importance of software citation. However, as explained in the abstract, the focus in fact is on providing “broadly applicable guidance on software citation”. My suggestion therefore is to revise the title. An alternative title for instance could be “How to cite software?”.

We agree with this comment, and have changed the title in response.

- “We recommend citing the specific version used (and the authors and publication date for that version) if you used it directly in the research described in your publication (e.g., the Methods section). We recommend citing the software concept (project) if you are referencing the software elsewhere in your paper.”: I don’t fully understand the distinction that is made in these two sentences. The authors seem to have in mind a distinction between citing software because it is used directly in a research project and citing software for other reasons. I would like to know more about what other reasons for citing software the authors have in mind and why they believe citations should be made in different ways in the two situations they distinguish.

We agree that this was not clear as written, and have rewritten these sentences.

- “If a published article exists that describes the software, it should be cited as an additional reference.”: The motivation for this recommendation is not clear to me. The authors seem to give special treatment to published articles, by which I assume they have in mind articles published in scholarly journals. I find this questionable. Suppose we have two pieces of software. Software A is documented in a two-page article published in a scholarly journal. Software B is documented in a comprehensive report made available in GitHub. Why should the article documenting software A be cited, while the report documenting software B does not need to be cited? Note that the article documenting software A probably cannot be updated, and the article is therefore likely to provide an outdated description of the software. The report documenting software B can be updated and therefore is likely to offer an up-to-date description of the software.

We have removed "published", as this was not an important part of the point we were trying to make, and have adjusted the text to make our point more clearly.

- “Hardware is important, but we have initially chosen not to overload software citations with hardware requirements directly. This might be better done through linkage between DOIs.”: I don’t understand these two sentences. Some additional explanation would be helpful

This text made sense in a much earlier version of the paper, but now we agree that this point was confusing as written and also feel it is not important to the article, so we have removed it.
